# Flotillin-2 Modulates Fas Signaling Mediated Apoptosis after Hyperoxia in Lung Epithelial Cells

**DOI:** 10.1371/journal.pone.0077519

**Published:** 2013-10-18

**Authors:** Shuquan Wei, Hyung-Geun Moon, Yijie Zheng, Xiaoliang Liang, Chang Hyeok An, Yang Jin

**Affiliations:** 1 Division of Pulmonary and Critical Care, Department of Medicine, Brigham and Women’s Hospital, Harvard Medical School, Boston, Massachusetts, United States of America; 2 Guangzhou First People's Hospital, Guangzhou Medical University, Guangzhou, China; University of Pittsburgh, School of Medicine, United States of America

## Abstract

Lipid rafts are subdomains of the cell membrane with distinct protein composition and high concentrations of cholesterol and glycosphingolipids. Raft proteins are thought to mediate diverse cellular processes including signal transduction. However, its cellular mechanisms remain unclear. Caveolin-1 (cav-1, marker protein of caveolae) has been thought as a switchboard between extracellular matrix (ECM) stimuli and intracellular signals. Flotillin-2/reggie-1(Flot-2) is another ubiquitously expressed raft protein which defines non-caveolar raft microdomains (planar raft). Its cellular function is largely uncharacterized. Our novel studies demonstrated that Flot-2, in conjunction with cav-1, played important functions on controlling cell death *via* regulating Fas pathways. Using Beas2B epithelial cells, we found that in contrast to cav-1, Flot-2 conferred cytoprotection *via* preventing Fas mediated death-inducing signaling complex (DISC) formation, subsequently suppressed caspase-8 mediated extrinsic apoptosis. Moreover, Flot-2 reduced the mitochondria mediated intrinsic apoptosis by regulating the Bcl-2 family and suppressing cytochrome C release from mitochondria to cytosol. Flot-2 further modulated the common apoptosis pathway and inhibited caspase-3 activation *via* up-regulating the members in the inhibitor of apoptosis (IAP) family. Last, Flot-2 interacted with cav-1 and limited its expression. Taken together, we found that Flot-2 protected cells from Fas induced apoptosis and counterbalanced the pro-apoptotic effects of cav-1. Thus, Flot-2 played crucial functions in cellular homeostasis and cell survival, suggesting a differential role of individual raft proteins.

## Introduction

Apoptosis and necrosis are well known as two traditional cell death processes [1], [2]. Many noxious stimuli induce either apoptosis or necrosis or both, depending on the cell type, the strength of stimulation and the presence of apoptosis inhibitors [3]–[5]. Cell surface receptors are responsible to transmit “death” signals from extracellular milieu to intracellular compartments and evoke a cascade of intracellular responses. Fas (CD95/APO-1/TNFRSF6) is one of these cell surface receptors and belongs to the tumor necrosis factor (TNF) receptor superfamily [6]. Fas has been reported to mediate both the caspase-dependent apoptotic death and the caspase-independent necrotic death [7]. Both pathways are regulated through Fas associated death domain (FADD). Fas-mediated apoptosis is transmitted through two pathways, caspase-8 associated extrinsic apoptotic pathway and mitochondria dependent intrinsic pathway [8], [9]. After exposure to Fas ligand (FasL) or other noxious stimuli including oxidative stress [10], Fas undergoes a conformational change to expose its death domain. Other death domain-containing proteins, such as FADD, interact with Fas [11]–[13] and promote the assembly of the death-inducing signaling complex (DISC)[14]. The components in DISC include not only Fas and FADD, but also the ‘death effector domain’ (DED) -containing procaspase-8. Cysteine proteases are concentrated in the DISC assembly and subsequently induce auto-proteolytic cleavage of caspase-8. Activations of caspase cascades are initiated at different points, such as at the plasma membrane/cytosol (DISC formation, extrinsic pathway) or at the mitochondria (intrinsic pathway) [10], [12]–[14]. Intrinsic pathway can be stimulated by viral infections, toxins, free radicals or damage to DNA. These stimuli induce the loss of mitochondrial transmembrane potential, leading to the release of proapoptotic proteins into the cytosol [15]. The mitochondrial (intrinsic) pathway is initiated by the release of pro-apoptotic proteins from the mitochondria to cytosol, including cytochrome *c*, apoptosis5 inducing factor (AIF), second mitochondria-derived activator of caspase (Smac)/direct inhibitor of apoptosis protein (IAP)-binding protein with low PI (DIABLO) etc. [16], [17]. Cytosolic cytochrome *c* induces caspase-3 activation *via* the apoptosome complex. During this process, Smac/DIABLO counters the inhibitory effects of the IAPs and thus promotes caspase-3 activation [17].

Unlike apoptosis, caspase activation is not required for necrosis [18], [19]. However, shown in previous reports, Fas may activate both apoptotic and necrotic pathways, *via* FADD [7], [20]. Oxidative stress has also been shown to induce cell death *via* both apoptosis and necrosis [4], [10], [21]. H2O2 and –OH are crucial components in Fas-induced cell death. Involvement of Fas usually causes apoptosis. When Fas activation prolongs, the cells proceed to necrotic cell death [22]. Further, FADD has also been demonstrated to engage in the necrotic death signaling in caspase-8–null cells [22].

Oxidative stress and the generation of reactive oxygen species (ROS) are important culprits for lung epithelial death and lung injury [23]. However, the detailed cellular mechanisms involved in the oxidative stress induced cell death remain not completely understood, thus, therapeutic targets are limited. Previous reports have shown that upon stimulation, cell surface receptor Fas migrates into lipid rafts to achieve the formation of DISC [24], [25]. Fas interacts caveolin-1 (cav-1), the marker protein of caveolae, after exposure to the ROS which is derived from either hyperoxia or cigarette smoking [26], [27]. Cav-1 functions as a platform on which the Fas mediated DISC formation is accomplished [26], [27].

Lipid rafts are subdomains of the cell membrane which contain distinct protein composition and high concentrations of cholesterol and glycosphingolipids [28]. Two types of lipid rafts have been reported including planar rafts and caveolae. Flotillin-2/formerly called reggie-1 (Flot-2) is a highly conserved lipid raft marker protein initially identified in murine lung tissue [29], [30]. In contrast to cav-1 which resides in the omega shaped caveolae, Flot-2 mainly locates in the planar portion of lipid rafts and has an amino acid identity of 99% between mouse and man, 61% between mouse and *Drosophila,* indicating its important cellular functions [29], [30]. Although richly expressed in almost all the pivotal cells and tissue, the exact cellular functions of Flot-2 are poorly understood. In our current studies, we for the first time, identified that Flot-2 plays a crucial role in regulating Fas mediated signaling. Interestingly, Flot-2 seems to counterbalance the effect of cav-1. Thus, our study revealed a novel insight on how lipid raft proteins help to maintain the cellular homeostasis and cell survival, in the presence of a death signal.

## Materials and Methods

### Reagents and chemicals

Flot-2 antibodies were purchased from Sigma (St. Louis, MO). All other antibodies were obtained from Santa Cruz Biotechnology (Santa Cruz, CA) or Cell Signaling (Danvers, MA). Flot-2 small interfering RNAs (siRNAs) and overexpression clones were from Santa Cruz and Genecopoeia (Rockville, MD) respectively. Immunohistochemistry reagents and kit were purchased from Vectors Laboratories (Burlingame, CA). Taqman PCR kit was purchased from Applied Biosystems (Foster City, CA). All other reagents and chemicals were from Sigma.

### Animal Studies

Cav-1–/– mice, and matched wild type C57BL/6 mice (male, 6 to 8 weeks of age), were obtained from Jackson Lab (Maine, USA). All mice were maintained under specific pathogen-free conditions and all mice experiments were performed in compliance with the Guide for the Care and Use of Laboratory Animals published by the US National Institutes of Health (NIH) and the guidelines of the Harvard Medical Laboratory Animal Care and Use Committee. The full name of IACUC who approved the protocol is “Harvard Medical Laboratory Animal Care and Use Committee”. Method of euthanasia:

Mice were euthanized in a carbon dioxide chamber. Primary alveolar epithelial cells were isolated from the above mentioned mice. Briefly, mouse lungs were washed with 20 ml PBS, followed by 2 ml dispase and 0.5 ml Agarose. Lungs were dissociated in DMEM with 25 mM HEPES and 200 U/ml DNase. Cells were then plated on the CD45 and CD16/32 coated dishes. After centrifuge, the pellets were re-suspended in DMEM with 10% FBS.

### Cell culture

Beas2B cells were obtained from the ATCC (Manassas, VA). Beas2B cells were cultured in DMEM medium with 10% FBS (GIBCO, NY). All cells were grown at 37°C in a humidified atmosphere of 5% CO2–95% air. For hyperoxia treatment, cells were exposed to hyperoxia (95% oxygen with 5% CO2) in modular exposure chambers.

### Cell viability assay and Caspase 3/7 activity assay

CellTiter-Glo Luminescent Cell Viability Kit, CellTiter-Blue® Cell Viability and Caspase-Glo 3/7 Assay kit were purchased from Promega (Madison, WI). Briefly, equal number cells were seeded in each well using 96-well plate. Transfection was performed and then cells were treated with hyperoxia (95% oxygen and 5% carbon dioxide) for designated time. Before measuring, cells were washed twice with PBS. 100 µl CellTiter-Glo reagent or Caspase-Glo 3/7 reagent and 100 µl PBS were added to each well and then incubated for 30 mins at room temperature. The luminescent signal was measured using the FLx800 Fluorescence Microplate Reader (BioTeck, Winooski, VT).

### Western blot analysis

Cells were harvested after twice cold PBS washing and then re-suspended in lysis buffer (RIPPA buffer) with protease inhibitors (Roche, Indianapolis, IN). Total protein samples were resolved by 4–12% NuPAGE gel (Invitrogen, Carlsbad, CA ) and transferred to nitrocellulose membranes (Bio-Rad, Hercules, CA). Membranes were blocked in 5% nonfat milk in PBST for 1 hour at room temperature and then blocked with primary antibodies at 4°C overnight. Membranes were washed and incubated with appropriate secondary antibodies (Santa Cruz, CA). Detection was performed using the SuperSignal West Pico system (Pierce, IL) and exposed to x-ray film (FUJIFILM, Japan).

### RNA extraction, qRT-PCR

Total RNA of cultured cells was extracted with TRIzol reagent. cDNA was synthesized using 1 µg of total RNA per sample with ABI mRNA reverse transcription kit (Applied Biosystems, CA), according to the manufacturer’s manual. Quantitative reverse transcription-PCR was performed in triplicate for each sample using the TaqMan Probe-Based Gene Expression Analysis according to the manufacturer’s instructions (Applied Biosystems, CA).

### Confocal microscopy

Briefly, cells were collected and fixed with 4% paraformaldehyde for 30 minutes and then permeabilized by 0.5% TritonX-100 for 1 hour at room temperature. Cells were blocked with 5% BSA in PBS for 2h at room temperature before incubating with primary antibodies at 4°C overnight. Cells were then washed three times and secondary fluorescein-conjugated antibodies (Santa. Cruz, CA) were applied at 1:200 dilution at room temperature for 1 hour. Images were captured using Olympus Fluoview BX 61 confocal microscope (Olympus, Center Valley, PA).

### Immuno-histo-Chemistry (IHC)

Immunohistochemistry was performed per the manufacturer's instruction using Vectastain Universal

Elite ABC kit (VECTOR LABORATORIES, CA). Briefly, Paraffin-embedded sections were incubated at 60°C for 1 hour and then de-waxed with xylene. Sections were rehydrated in serial ethanol solutions and then immersed in the re-heated retrieval solution for 30 minutes at 95°C. Endogenous peroxidases were blocked by incubating sections in 0.3% H_2_O_2_ for 30 minutes. Sections were pre-blocked with normal donkey serum before being incubated with primary antibodies at 4°C overnight. The immuno-reactions were visualized with peroxidase substrate. Nucleus was stained with Hematoxylin QS.

### Co-immunoprecipitation (Co-IP)

Cells were washed twice with PBS and then re-suspended in lysis buffer with protease inhibitor mixtures (Roche, Indianapolis, IN). The samples were centrifuged and the supernatant were incubated with primary antibodies at 4°C overnight. Protein A/G Sepharoses (Santa Cruz, CA) were incubated with samples for an additional 3 hours at 4°C. Immune complexes were precipitated by centrifugation.

### Statistical Analysis (Anova)

To test the differences among independent samples, the means of fold change in all Figures were compared using two-way analysis of variance. When p< 0.05, the difference was considered statistically significant. Error bars indicate the standard deviation.

## Results

### Deletion of Flot-2 promoted cell death and caspase-3 dependent apoptosis

**Figure 1:**
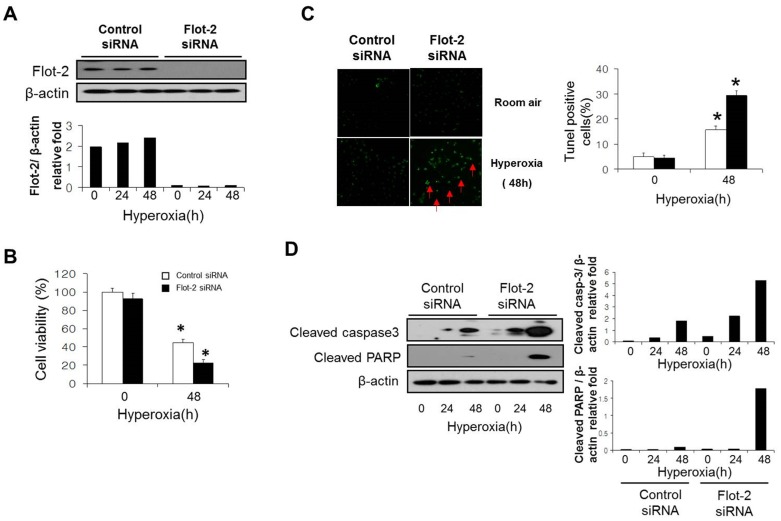
Silencing of Flot-2 promoted cell death and caspase-3 dependent apoptosis. (A) Beas 2B cells were transfected with Flot-2 siRNA and control siRNA. After 24h, cells were treated with hyperoxia (95% oxygen) or room air. To determine the transfection efficiency, cell lysate was subjected to Western Blot Analysis using anti-Flot-2 antibodies. (B) After another 48h, cell viability was performed using CellTiter-Glo Luminescent Cell Viability Assay kits (Promega). (C) TUNEL staining of Flot-2 silenced Beas 2B cells after hyperoxia. Cells were first transfected with Flot-2 siRNA and control siRNA. As above, cells were exposed to hyperoxia and room air. After 48h, TUNEL staining was performed as described in materials and methods. Left panels: representative images of the TUNEL staining. Apoptotic nuclei were labeled by TUNEL (green, red arrow). Right panel: frequency of TUNEL-positive cells. (D) Deletion of Flot-2 promoted caspase-3 dependent apoptosis. Flot-2 silenced Beas 2B and control cells were exposed to hyperoxia or normoxia (room air) for the indicated time. Cells were harvested and cell lysate was subjected to Western Blot Analysis. Cleaved caspase-3 and cleaved PARP were detected using antibodies specific for the cleaved (active) forms. All the above figures represented three independent repeats with similar results. *P<0.05.

In our previously work, we observed that deletion of cav-1 (cav-1^−/−^) protects against hyperoxia induced apoptosis in lung epithelial cells [26]. In current study, we investigated the cellular function of reggie1/Flot-2, another lipid raft protein thought to locate in planar rafts. Initially, using immunohistochemistry (IHC) staining, we found that Flot-2 expressed ubiquitously in mouse lung, including both alveolar epithelial cells and bronchial epithelial cells (Fig. S1). Lung epithelial cell death is a prominent feature involved in oxidative stress induced acute lung injury [10], [21], [26], [31]. To determine the effects of Flot-2 on epithelial cell death, Beas2B human epithelial cells were transfected with Flot-2 siRNA to achieve the deletion (Fig. 1A). After exposure to hyperoxia (48h), we found that deletion of Flot-2 promoted hyperoxia-induced cell death, determined using CellTiter-Glo Luminescent Cell Viability Assay (Fig. 1B). TUNEL staining further confirmed that silencing of Flot-2 enhanced hyperoxia induced apoptosis (Fig. 1C). Additionally, deletion of Flot-2 robustly increased hyperoxia induced caspase-3 and Poly (ADP-ribose) polymerase (PARP) activation indicated by increased cleaved (active) forms, using Western Blot Analysis (Fig. 1D). Further, using human Beas 2B cells, we assessed the expression of Flot-2 in the presence and absence of hyperoxia. After a short exposure of hyperoxia (<24h), there is no significant changes on Flot-2 expression (data not shown).

### Flot-2 directly interacted with Fas pathway and prevented DISC formation

**Figure 2:**
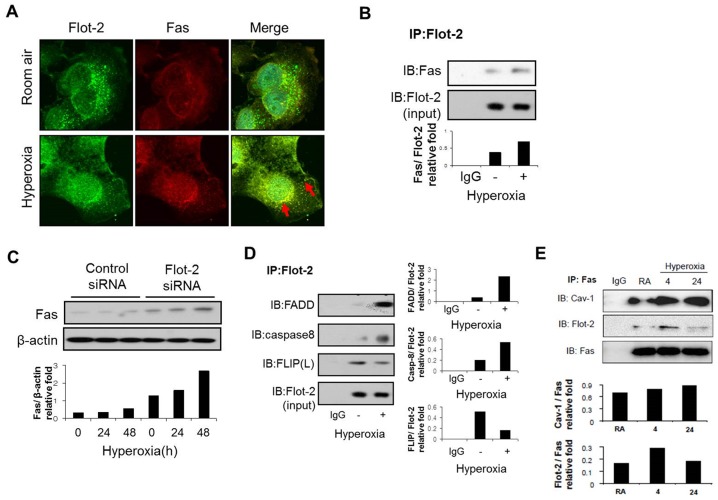
Interactions between Flot-2 and the components of DISC. (A) Co-localization of Flot-2 and Fas after hyperoxia. Beas 2B cells were exposed to hyperoxia or room air as described previously. After 4h, the cells were stained with anti-Flot-2 and anti-Fas respectively. Cells were then examined using confocal microscopy. Flot-2 (green), Fas (red), merge (yellow, red arrow). (B) Interactions between Flot-2 and Fas after hyperoxia. Beas-2B cells were exposed to hyperoxia or room air. After 4h, cells were collected and cell lysate was subjected to co-IP. Samples were first immunoprecipitated with anti-Flot-2 antibody. Western Blot Analysis was performed subsequently using anti-Flot-2 and anti-Fas. (C) Silencing of Flot-2 up-regulated the expression of Fas. Beas 2B cells were transfected with Flot-2 siRNA or control siRNA. After hyperoxia, cell lysate was subjected to Western Blot Analysis with anti-Fas. (D) Interactions between Flot-2 and other components of DISC. Beas-2B cells were exposed to hyperoxia or room air. After 4h, cells were collected for co-IP assays. Samples were first immunoprecipitated with anti-Flot-2. Next, FADD, caspase-8, FLIP antibodies were used to determine the interactions respectively using Western Blot Analysis. (E) Protein-protein interaction assayed by Co-IP. Beas2B cells were exposed to hyperoxia (4 and 24 h) and room air. Cell lysate was immunoprecipitated with anti-Fas, then Western Blot Analysis was performed and membranes were incubated with anti-Flot-2, anti-Fas and anti-Cav-1 respectively All figures represented three independent repeats with similar results.

**Figure 3:**
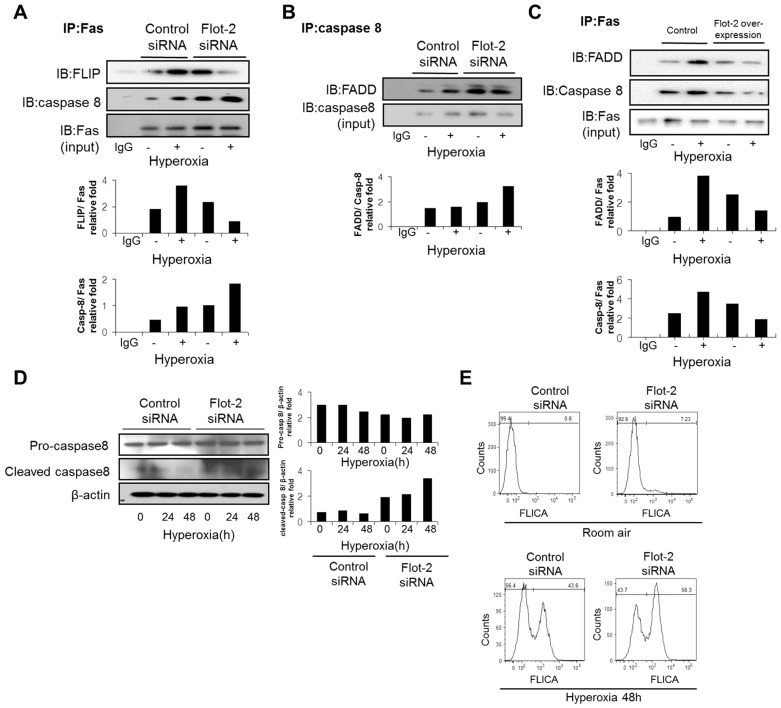
Deletion of Flot-2 enhanced DISC formation and caspase 8 mediated extrinsic apoptosis. (A) - (B): Beas 2B cells were transfected with Flot-2 siRNA or control siRNA, and exposed to hyperoxia or room air. After 4h, cells were harvested and subjected to Co-IP. (A) Samples were immunoprecipitated with anti-Fas antibodies, followed by Western Blot using anti-FLIP, caspase-8 and Fas antibodies respectively. (B) Samples were first immunoprecipitated with anti-caspase 8 antibodies, followed by Western Blot using anti-FADD antibodies. (C) Over-expression of Flot-2 inhibited DISC formation: Flot-2 was over-expressed in Beas 2B cells. Empty vectors were used as controls. Transfected cells were then exposed to hyperoxia or room air. After hyperoxia (4h), cells were harvested and subjected to Co-IP assays. Samples were first immunoprecipitated with anti-Fas antibodies, followed by Western Blot Analysis using anti-FADD, caspase-8 and Fas antibodies respectively. (D) Deletion of Flot-2 up-regulated the cleaved caspase-8. Beas 2B cells were transfected with Flot-2 siRNA and control siRNA, and exposed to hyperoxia or room air for the indicated time. Both total and cleaved caspase-8 was detected. (E) Deletion of Flot-2 enhanced caspase-8 activation after hyperoxia. Flot-2 silenced Beas 2B cells were exposed to hyperoxia and room air. After 48h, cells were collected and caspase-8 activity was analyzed using flow cytometry and Vybrant® FAM Caspase-8 Assay Kit as described in material and methods. All figures above represented three independent assays with similar results.

Death-inducing signaling complex (DISC) is important in death-receptor-mediated apoptosis in epithelial cells after oxidative stress [10]. We first determined whether Flot-2 co-localizes with Fas in Beas2B epithelial cells. As shown in Fig. 2A, Flot-2 co-localized with Fas, particularly robust after exposure to hyperoxia (4h) (Fig. 2A). Co-IP assays confirmed this interaction between Flot-2 and Fas (Fig. 2B and Fig 2E). Fas and Flot-2 interactions increased shortly after hyperoxia (4h) (Fig. 2B). However, prolonged hyperoxia diminished the binding between Fas and Flot-2 (>24h) (Fig 2E). Interestingly, Flot-2 not only physically interacted with Fas, but also was required for Fas protein expression. Deletion of Flot-2 up-regulated the hyperoxia induced Fas expression in a time dependent manner, using Western Blot Analysis (Fig. 2C). Furthermore, we observed that Flot-2 may transport to the nucleus after hyperoxia, observed using GFP-labeled Flot-2 (Fig S2). We next investigated the interactions between Flot-2 and other DISC components including FADD, caspase-8 (FLICE) and FLICE-like inhibitory protein (FLIP), in the absence and presence of hyperoxia. We found that hyperoxia induced the interactions between Flot-2 and caspase-8 or FADD (Fig. 2D). On the other side, the interactions between Flot-2 and FLIP decreased after hyperoxia (Fig. 2D). FLIP inhibits caspase-8 and FADD, thus prevents apoptosis [32]. Our data showed that hyperoxia exerted an opposite effect on the interactions between Flot-2 and FADD/caspase-8 comparing to those between Flot-2 and FLIP. This result is consistent with previous reports on the inhibitory effects of FLIP on DISC formation. To determine the effects of Flot-2 on DISC formation, co-IP assays were used to determine the interactions among DISC components in wild type cells and Flot-2 deleted cells (silencing of Flot-2) in the absence and presence of hyperoxia. The interactions of Fas/caspase-8 or FADD/caspase-8 significantly increased in Flot-2 silenced cells after hyperoxia (Fig 3A, B), comparing with those in wild type cells. In contrast, the interactions between Fas and FLIP decreased in Flot-2 silenced cells (Fig. 3A). Additionally, we used the “gain of function” approaches to confirm the above. Over-expression of Flot-2 in Beas 2B cells resulted in decreased interactions between Fas/FADD and Fas/caspase8, comparing with those in empty vector transfected cells (Fig 3C).

### Flot-2 affected caspase-8 mediated extrinsic apoptotic pathway

As above, Flot-2 was required to prevent DISC formation (Fig 3A-C), indicating a role in the regulations of extrinsic apoptotic pathway. We confirmed this hypothesis by determining the caspase-8 activation using Western Blot Analysis and Flow Cytometry. Silencing of Flot-2 promoted the hyperoxia-induced cleavage of caspase-8 (active form) and caspase-8 activities (Fig 3D, E).

### Flot-2 affected the mitochondria dependent, intrinsic apoptotic pathway

**Figure 4:**
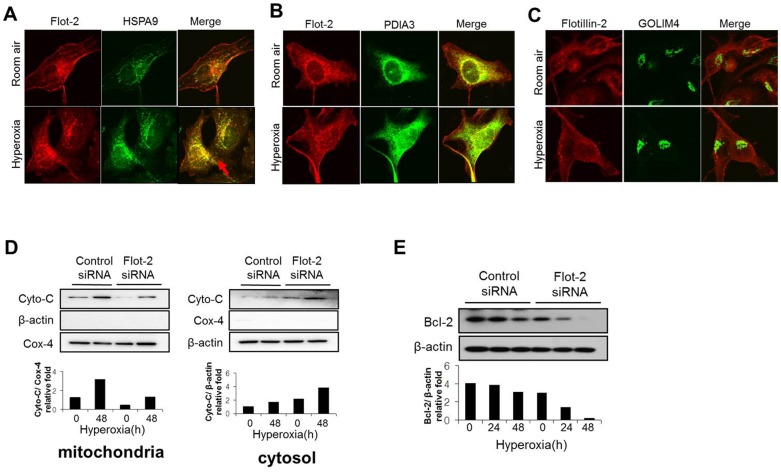
Deletion of Flot-2 enhanced mitochondria mediated intrinsic apoptosis. (A): Intracellular location of Flot-2, in the presence and absence of hyperoxia. Beas 2B cells were exposed to room air or hyperoxia, as described previously. After 4h, cells were stained with anti-Flot-2 and anti-HSPA9 (markers of Mitochondria). Next, the cells were observed under confocal microscopy. Red: Flot-2; Green: HSPA9; Yellow: merge. (B): Beas 2B cells were exposed to room air or hyperoxia, as described previously. After 4h, cells were stained with anti-Flot-2, anti-PDIA3 (markers of ER). Next cells were observed under confocal microscopy. Red: Flot-2; Green: PDIA3 for ER, Yellow: merge (C): Beas 2B cells were exposed to room air or hyperoxia, as described previously. After 4h, cells were stained with anti-Flot-2, anti- GOLIM4 (markers of Golgi) and then cells were observed under confocal microscopy. Red: Flot-2; Green: GOLIM4 for Golgi; Yellow: merge (D) Silencing of Flot-2 regulated cytochrome C (Cyto-C) release from mitochondria to cytosol, after hyperoxia. Beas-2B cells were transfected with Flot-2 siRNA or control siRNA. After hyperoxia or room air (48h), mitochondria and cytosol were isolated from these cells using Mitochondria Isolation Kit, as described in material and methods. Equal amount of mitochondria and cytosol were used to perform Western Blot. Left panel: Cyto-C detected in mitochondria; Cox-4 was used as the mitochondria marker and β-actin as the cytosol marker. Right panel: Cyto-C detected in cytosol. (E) Silencing of Flot-2 down-regulated the expression of Bcl-2. Beas 2B cells were transfected with Flot-2 siRNA or control siRNA. Cells were next exposed to hyperoxia or room air. After the indicated time courses, the whole cell lysate was subjected to Western Blot Analysis using anti-Bcl-2. All figures above represented three independent experiments with similar results.

Despite initially identified on cell plasma membrane, Flot-2 potentially locates also in intracellular organelles and nucleus given its critical cellular functions observed. To first determine the distribution of Flot-2 in cell organelles, we evaluated the co-localization of Flot-2 and three major organelle markers. We used anti-HSPA9, PDIA3 and GOLIM4 antibodies, as the markers for Mitochondria, ER and Golgi apparatus respectively. We observed that Flot-2 localized in Mitochondria and ER, but not significantly in Golgi apparatus (Fig. 4A, 4B, 4C). After hyperoxia, the co-localization between Flot-2 and HSPA9 (marker for mitochondria) increased significantly (Fig. 4A). In contrast, no significant increase of the co-localization between Flot-2 and PDIA3 (ER markers), indicating a specific role of flot-2 in mitochondria dependent apoptosis (Fig. 4B,C).

We next evaluated the Cytochrome-C (Cyto-C) release from mitochondria, an important component mediating intrinsic apoptotic pathway. Mitochondria were isolated from wild type Beas 2B cells and

Flot-2 silenced cells in the presence and absence of hyperoxia. The expression of Cyto-C in both mitochondria and cytosol was analyzed using Western Blot Analysis. Silencing of Flot-2 decreased Cyto-C in mitochondria after hyperoxia (48h). Consistently, the release of Cyto-C in cytosol increased significantly in Flot-2 silenced cells (Fig. 4D). In addition, deletion of Flot-2 down-regulated the expression of Bcl-2 after hyperoxia in a time-dependent manner (Fig. 4E), further suggesting an important role of Flot-2 in intrinsic apoptosis.

### Deletion of Flot-2 down-regulated the expression of IAP families and up-regulated cytosolic SMAC

**Figure 5:**
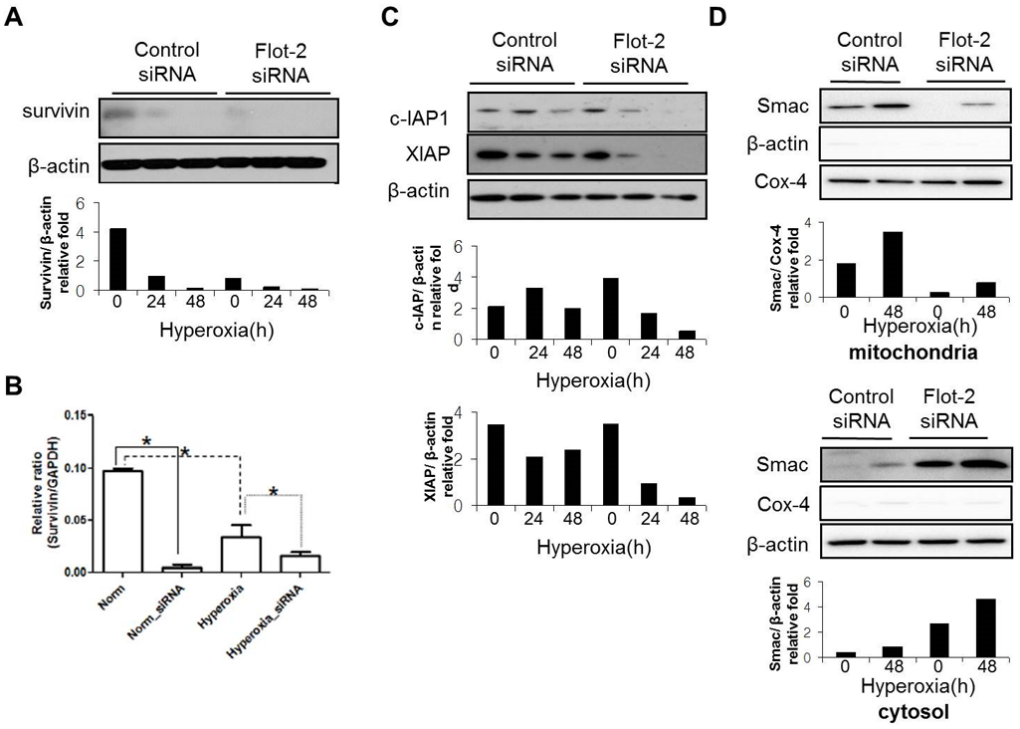
Silencing of Flot-2 down-regulated the expression of IAP family and up-regulated Smac. Beas 2B cells were transfected with Flot-2 siRNA or control siRNA. Cells were then exposed to hyperoxia or room air. After 24h or 48h, cell lysates were collected for Western Blot Analysis. (A) Protein expression level of survivin. (B) mRNA level of survivin determined by real-time PCR (C) Expression of c-IAP1 and XIAP (D) Silencing of Flot-2 regulated Smac release from mitochondria to cytosol after hyperoxia. As above, Beas 2B cells were transfected with Flot-2 siRNA or control siRNA. After hyperoxia (48h), mitochondria and cytosol were isolated using Mitochondria Isolation Kit as described in material and methods. Equal amount of mitochondria and cytosol were used to perform Western Blot Analysis. Upper panel: Smac detected in mitochondria; Cox-4 was used as the mitochondria marker and β-actin as the cytosol marker. Lower panel: Smac detected in cytosol. All figures above represented three independent experiments with similar results. *P<0.05.

Caspase-3 mediates the “common” apoptotic pathway and the activated caspase-3 leads to unavoidable cell death [2], [5]. However, there are inhibitors which counterbalance this last step of apoptotic pathway. IAP family proteins interact with caspase-3 and directly inhibit its activation [33], [34]. To complete our investigation on Flot-2, we next evaluated the effects of Flot-2 on IAP family members. Deletion of Flot-2 decreased the expression of survivin at both basal level and after hyperoxia in a time-dependent manner, determined using Western Blot Analysis and Real-time PCR (Fig. 5A and B). Furthermore, silence of Flot-2 down-regulated the expression of XIAP and c-IAP1 after hyperoxia (Fig. 5C). Smac/DIABLO binds with IAPs, and subsequently suppresses the function of IAPs (IAPs are caspase 3 inhibitors) [33], [34]. These interactions between Smac/DIABLO and IAPs occur in cytosol [33], [34]. Thus, with more cytosolic Smac/DIABLO, Smac/IAP interactions will potentially increase and less IAP will be available to inhibit caspase-3 activity. Interestingly, deletion of Flot-2 down-regulated the level of Smac/DIABLO in mitochondria after hyperoxia (48h), while the release of Smac/DIABLO into cytosol increased significantly (Fig. 5D).

### Deletion of Flot-2 promoted apoptosis partially *via* ROS generation

**Figure 6:**
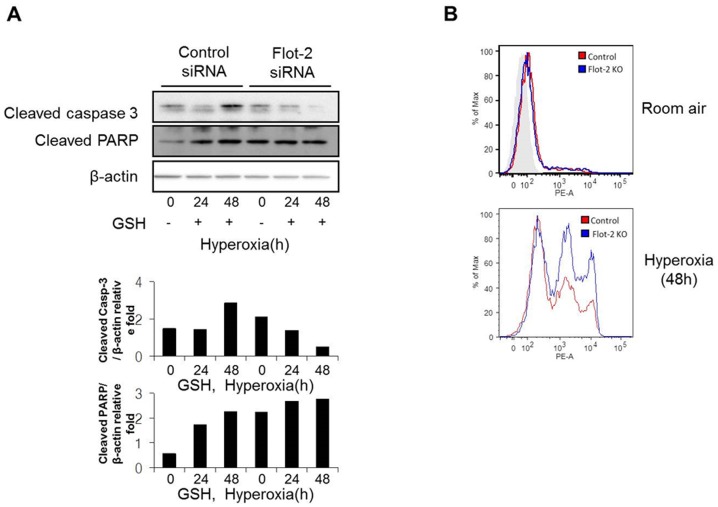
Deletion of Flot-2 promoted mitochondria mediated ROS generation. (A) Antioxidant diminished the effects of Flot-2-silencing. Beas2B cells were transfected with Flot-2 siRNA and control siRNA. Next, cells were treated with antioxidant glutathione (GSH, 80 µM) and exposed to hyperoxia. After 48h, cell lysate was subjected to Western Blot Analysis. (B) Deletion of Flot-2 in lung epithelial cells facilitated mitochondrial superoxide production after hyperoxia. Beas 2B cells were transfected with Flot-2 siRNA or control siRNA. After hyperoxia (0h, 24h or 48h), 2.5 mM MitoSox Red reagents were added to medium and incubated for 10 minutes at 37°C. Next, cells were analyzed using flow cytometer as described in material and methods. All figures above represented three independent experiments with similar results.

To determine whether ROS generation is one of the culprits to promote the silencing of Flot-2 mediated apoptosis, Beas 2B cells were treated with GSH (glutathione), a ROS scavenger, followed by hyperoxia. The silencing of Flot-2 induced cell death after hyperoxia was abolished in the presence of GSH (Fig. S3). Similarly, the hyperoxia induced up-regulation of cleaved caspase-3 and PARP in Flot-2 silenced cells was also blunted after adding GSH (Fig. 6A). Furthermore, deletion of Flot-2 generated higher amounts of mitochondrial superoxide anion radical, detected by MitoSOX fluorescence, suggesting an elevated mitochondrial ROS production in Flot-2 silenced cells (Fig. 6B).

### Deletion of Flot-2 augmented cav-1 expression

Our previous reports have shown a crucial role of cav-1 in regulating Fas mediated apoptosis [26].

**Figure 7:**
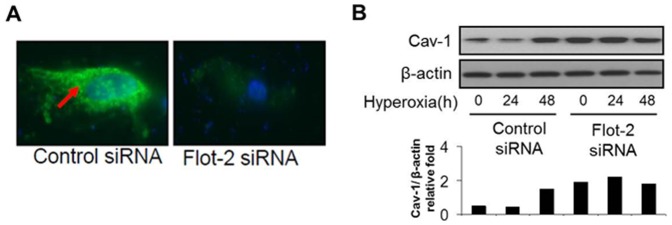
Silencing of Flot-2 up-regulated cav-1 expression. (A) Beas 2B cells were transfected with Flot-2 siRNA or control siRNA. Cells were then stained with anti-Flot-2 and Fluorescein (FITC) conjugated antibodies. Cells were examined using the fluorescence microscopy to determine the transfection efficiency. (B) After 24h or 48h, cell lysates were collected for Western Blot Analysis. The figures represented three independent experiments with similar results.

To determine the potential effects of Flot-2 on cav-1 expression, we transfected the Beas2B cells with control siRNA and Flot-2 siRNA (Fig. 7A). We found that silencing of Flot-2 resulted in an elevated cav-1 level, in the presence and absence of hyperoxia (Fig. 7B). We next isolated the epithelial cells from wild type and cav-1^−/−^ mice. Flot-2 level was up-regulated in cav-1^−/−^ cells comparing with those in wild type cells (Fig. S4A). Consistently, over-expression of cav-1 using cav-1 adenovirus in Beas2B cells resulted in decreased Flot-2 (Fig. S4B).

## Discussion

Fas mediated death signaling has been well characterized in previous literature in both apoptosis and necrosis. Compartmentalization of Fas signaling is crucial in defining cellular outcomes and highly involves the assembly of lipid raft – localized complex [24]–[26], [35]. Fas multimerizes and forms a cytoplasmic complex along with FADD and caspase-8 (DISC) [24]–[26], [35]. Subsequently, this DISC complex initiates the cellular apoptotic machinery [35]. During this process, the internalization of death receptor containing complex plays important roles in apoptosis cascade. DISC has been shown to continue binding with the internalized death receptor in the internalized vesicles [35]. For Fas mediated complex, the internalization step triggers recruitment of large amounts of FADD and caspase-8 to the endosomes. The internalization of Fas and its associated protein complex favors apoptosis by delivering the activated “death signal complex” *via* an endosome. On the other side, inhibition of Fas receptor internalization engages it to induce pro-survival pathways [35]. Caveolae and cav-1 have been known to involve in the internalization of Fas receptor complex and promote subsequent cell death [24], [25], [35]–[38]. Generally, blockade of cav-1- mediated endocytosis diminishes cell apoptosis [36]–[38]. In another word, cav-1 dependent internalization is required to enhance apoptotic signaling [36]–[38]. However, a different opinion exists regarding the apoptosis associated with cav-1 and TNF-related apoptosis-inducing ligand (TRAIL) in hepatocytes[39]. This differential observation potentially can be explained by the different cell types and stimuli. Despite this discrepancy, majority of reports confirm that cav-1 dependent internalization of death receptor complex promotes cellular death signaling and induces cell death, *via* apoptosis or necrosis. This is particularly apparent in Fas mediated pathways [26]. Cav-1 is abundant in lung epithelial cells at normal status, particular the type I cells [40], [41]. Cav-1 pY14 phosphorylation and internalization occur frequently in the presence of oxidative stress [26], [42]. One question arisen here is why majority of lung epithelial cells do not undergo spontaneous apoptosis in spite of rich cav-1 protein in their cell membrane. Apparently, although abundant in lung epithelial cells, cav-1 only represents a small portion of lipid raft proteins. Besides cav-1, large amount of lipid rafts are rich in flotillins and other protein components. We hypothesized that there is a counterbalance of cav-1 in lipid rafts which plays not the same, but opposite cellular functions comparing to cav-1. In our current studies, we identified that Flot-2 is the lipid raft protein which counterbalances the function of cav-1.

**Figure 8:**
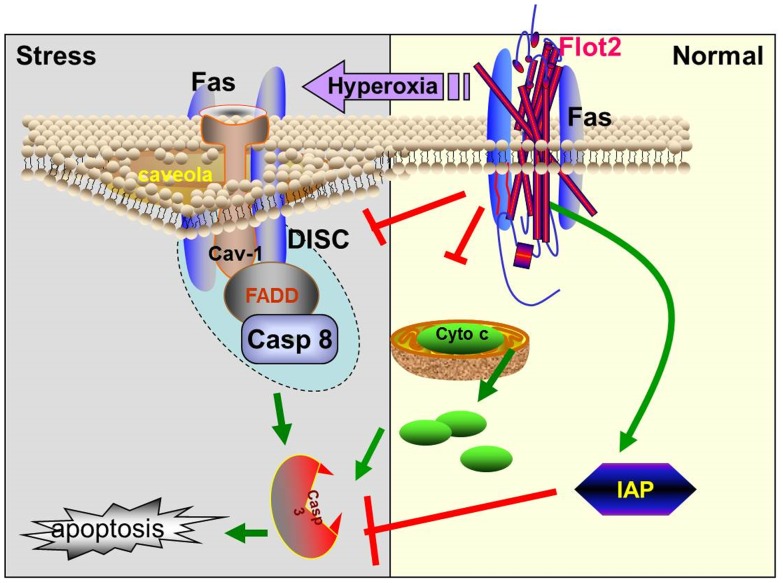
Schemata of proposed mechanisms. Flot-2, together with cav-1, maintains a homeostatic status and counterbalances the transduction of death signaling. At normal condition, Flot-2 interacts with Fas and blocks the contact of Fas and cav-1. The interaction of Flot-2 and Fas inhibits cav-1 mediated Fas aggregation and multimer formation in caveolae, subsequently inhibits the formation of Fas-dependent DISC complex along with FADD and caspase-8. Thus, Flot-2 acts as an antiapoptotic agent. After prolonged oxidative stress (>24h), such as hyperoxia, Flot-2 dissociates with Fas, resulting in an interaction between cav-1 and Fas. The binding between Fas and cav-1 leads to much more concentrated Fas in caveolae and promotes Fas multimerization, as well as the formation of DISC complex. Subsequently, the apoptosis cascade is triggered. Meanwhile, at normal conditions, mitochondria located Flot-2 prevents Cyto-C release from mitochondria to cytosol and also acts on the Smac/IAP family. All these effects of Flot-2 together add more weights to suppress intrinsic apoptosis and caspase-3 mediated common apoptotic pathways.

As shown in the schema of Fig. 8, Flot-2 interacts with Fas at normal status. The interaction of Flot-2 and Fas hence potentially inhibits the formation of Fas multimer and Fas-dependent DISC complex, subsequently suppresses the internalization of cav-1/Fas complex. Therefore, Flot-2 acts as an anti-apoptotic agent. Consistent with our hypothesis, at room air, Flot-2 silencing alone (without hyperoxia) also induces the interactions between Fas-caspase 8 and Fas-FADD (Fig 3A, B). A synergistic effect occurs after Flot-2 silencing in the presence of hyperoxia (Fig 3). In the presence of hyperoxia, the immediate response of the cells is to prevent Fas aggregation and try to save the cells from death. In order to prevent Fas multimerization, at the early time point (i.e., 4h after hyperoxia,

Fig 2A), there is an increased interaction between Fas and Flot-2. However, after prolonged hyperoxia (beyond 24h, Fig 2E), the interactions between Fas and Flot-2 saturate and decrease, indicating an unavoidable cell death. We believe that Flot-2 maintains a homeostasis of the lipid rafts and assists the cells to survive minor hyperoxic insult (short period). Prolonged hyperoxia results in an unsalvageable death. Similarly, Flot-2 silencing will lead to cell death due to the aggregation of Fas as explained above. After hyperoxia, the interactions between Fas and cav-1 lead to much more concentrated Fas level in caveolar portion of the rafts. Hence, Fas multimers are formed, as well as the DISC complex. Fas internalize with cav-1 in the endosomes and trigger the apoptosis cascade, as mentioned above.

Interestingly, we observed that Flot-2 locates not only on cell membrane, but also in cytosol, nucleus and organelles. It is very possible that Flot-2 internalizes but has different compartments with cav-1 dependent internalization, thus possesses different intracellular functions. For example, our studies show that Flot-2 regulates survivin protein level in an opposite direction comparing to cav-1[43]. One explanation to this observation is that Flot-2 interacts with cav-1 at one or multiple points along the gene regulatory pathways of survivin. Our previous studies have shown that cav-1 mediates not only extrinsic pathway, but also the intrinsic pathway *via* regulating the truncated BID during apoptosis [26]. Cav-1 has been found on endosome-like structures (caveosomes), endoplasmic reticulum (ER) and in mitochondria [36]-[39]. Further, recent reports indicate that cav-1 has a regulatory role in mitochondria associated oxidative stress [26], [44]. Certain anti-tumor agents promote a redistribution of lipid rafts from the plasma membrane to mitochondria and induce a raft-associated intrinsic apoptosis via mitochondria [45]. Interestingly, we found that Flot-2 also localizes on mitochondria. It remains not entirely clear how Flot-2 suppresses mitochondria mediated intrinsic apoptotic pathway and whether it counterbalances the effects of cav-1 in mitochondria. It is very possible that the re-distribution of Flot2 and/or other lipid raft proteins from plasma membrane to mitochondria plays crucial function on directing the fate of cells, either survival or death. Additionally, it will be more interesting to analyze the subcellular localization of both Cav-1 and Flot-2 under the different death inducers, such as LPS or TNF-alpha. These death signals may exert a completely different pattern of lipid raft distribution comparing with hyperoxia. These will be our future directions in further studies.

The co-localization of Flot-2 and ER is not surprising. Most transmembrane proteins are inserted into the ER membrane, so do the flotillins [46]. In fact, Morrow et al has reported that flotillins are transmembrane proteins associated with many organelles and play important roles in transporting [46].

Last, our results are consistent with the previous reports on the role of ROS as key participants in Fas induced cell death and apoptosis (Fig 6) [10].

Our study shows that Flot-2 counterbalances the effects of cav-1, at multiple points along the apoptotic pathways. However, this observation should not be extrapolated to the two types of lipid rafts. Although Flot-2 is thought to locate mainly in planar lipid rafts while cav-1 locates in caveolae, no definite techniques can differentiate these two types of lipid rafts directly [47], [48]. In fact, whether flotillins locate only in planar lipid rafts or locate in both types of rafts remain unclear [47], [48]. At least one report has shown that Flot-1 locates also in caveolae [49], despite that no reports focus on the Flot-2 yet.

Our current studies have raised some questions requiring future investigations. One of them is that we did not address the function and potential interaction between Flot-1 and Flot-2. Flot-1 and Flot-2 have about 40 to 50% identity [50]. Similar like Flot-2, Flot-1 is thought as a lipid raft marker protein. It may also play crucial and intriguing functions in maintaining cellular homeostasis along with all other lipid raft proteins.

In summary, our study is the first report illustrating a regulatory function of Flot-2 in Fas mediated apoptosis. It further reveals a novel insight on how lipid raft proteins participate in the regulation of cell survival and death. A complex of lipid raft proteins is composed of cav-1, Flot-2 and many more other members in the raft family. All these raft members coordinate and interact with each other to maintain a homeostatic status and balance the transduction of different signals derived from ECM. Therefore, raft protein members potentially carry either similar or opposite cellular functions, defining a distinct role in the signaling complex.

## Supporting Information

Figure S1 **Localization of Flot-2 in mice lung tissue. C57BL/6J mice were exposed to hyperoxia or room air.** After 0, 1, and 2 days, mouse lung tissue were obtained and stained with anti-Flot-2. Brown color: Flot-2 (black arrows).
(TIF)

Figure S2 **Localization of Flot-2-GFP in Beas2B cells.** Cells were exposed to room air and hyperoxia (4h) and the Flot-2 was observed under confocal microscopy. Red arrow: Flot-2.
(TIF)

Figure S3 **Antioxidant diminished the effects of Flot-2-silencing.** Beas 2B cells were transfected with Flot-2 siRNA and control siRNA. Next, cells were treated with antioxidant glutathione (GSH, 80 µM) and exposed to hyperoxia. After 48h, cell viability was performed using CellTiter-Glo Luminescent Cell Viability Assay as described in material and methods. All figures above represented three independent experiments with similar results. ns: non-significant.
(TIF)

Figure S4 **Deletion or enhancement of cav-1 regulates Flot-2 expression.** (A) Primary lung epithelial cells were isolated from wild type C57BL/6J mice or cav-1^−/−^ mice. Cells were then exposed to room air or hyperoxia. After 24h, cell lysate was collected and subjected to Western blot analysis. (B) LacZ and adeno-cav-1 transfected to Beas2B cells for 48h. And then Cells were then exposed to room air or hyperoxia for 24h. The figure represented three independent experiments with similar results.
(TIF)
