# Supplementary figures and images for: Flotillin-2 Modulates Fas Signaling Mediated Apoptosis after Hyperoxia in Lung Epithelial Cells
Source: PLoS One. 2013 Oct 18;8(10):e77519. doi: 10.1371/journal.pone.0077519 (PMC3799625; doi:10.1371/journal.pone.0077519)

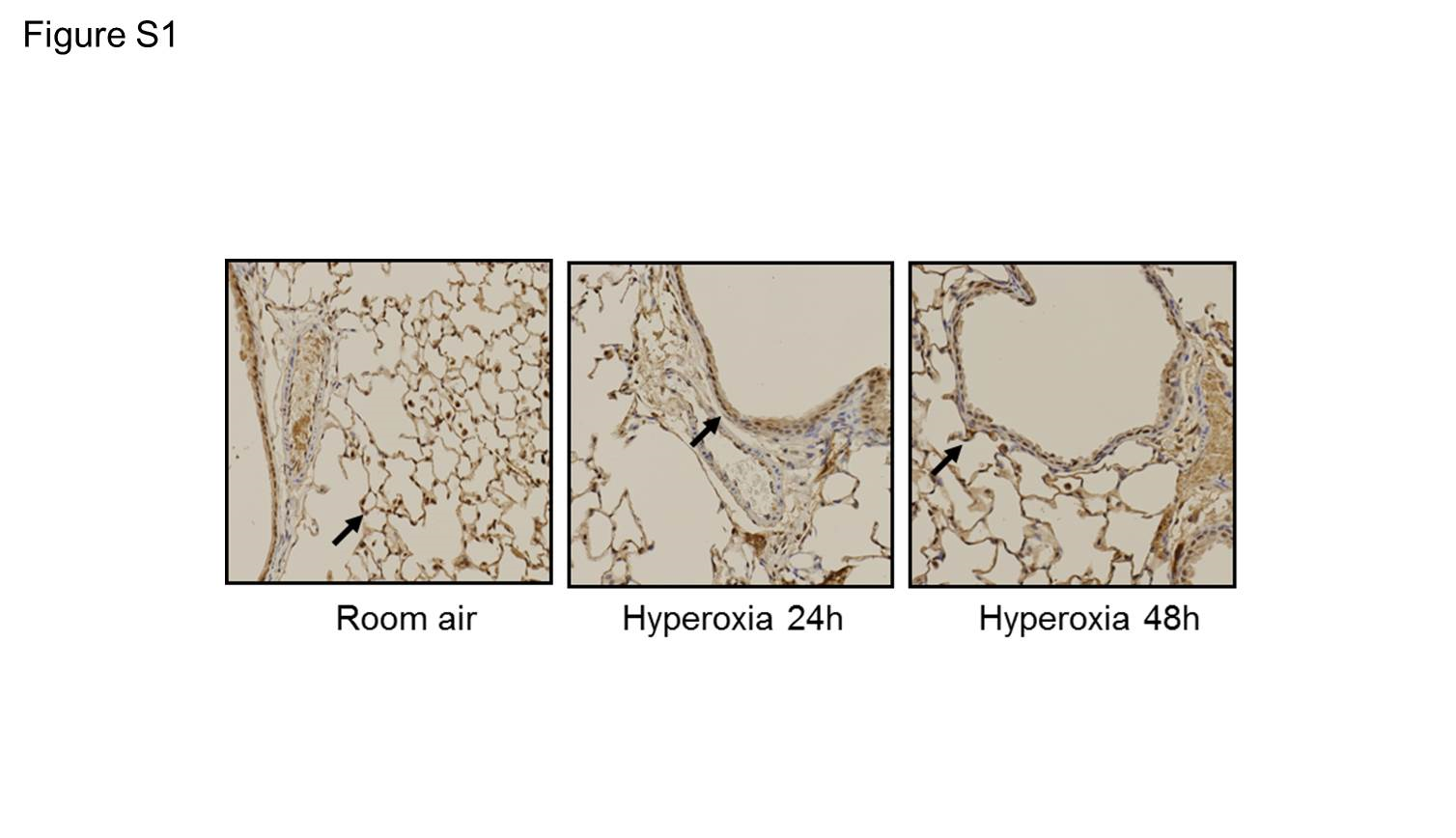

Supplement: Figure S1 — Localization of Flot-2 in mice lung tissue. C57BL/6J mice were exposed to hyperoxia or room air. After 0, 1, and 2 days, mouse lung tissue were obtained and stained with anti-Flot-2. Brown color: Flot-2 (black arrows). (TIF) [file pone.0077519.s001.tif]

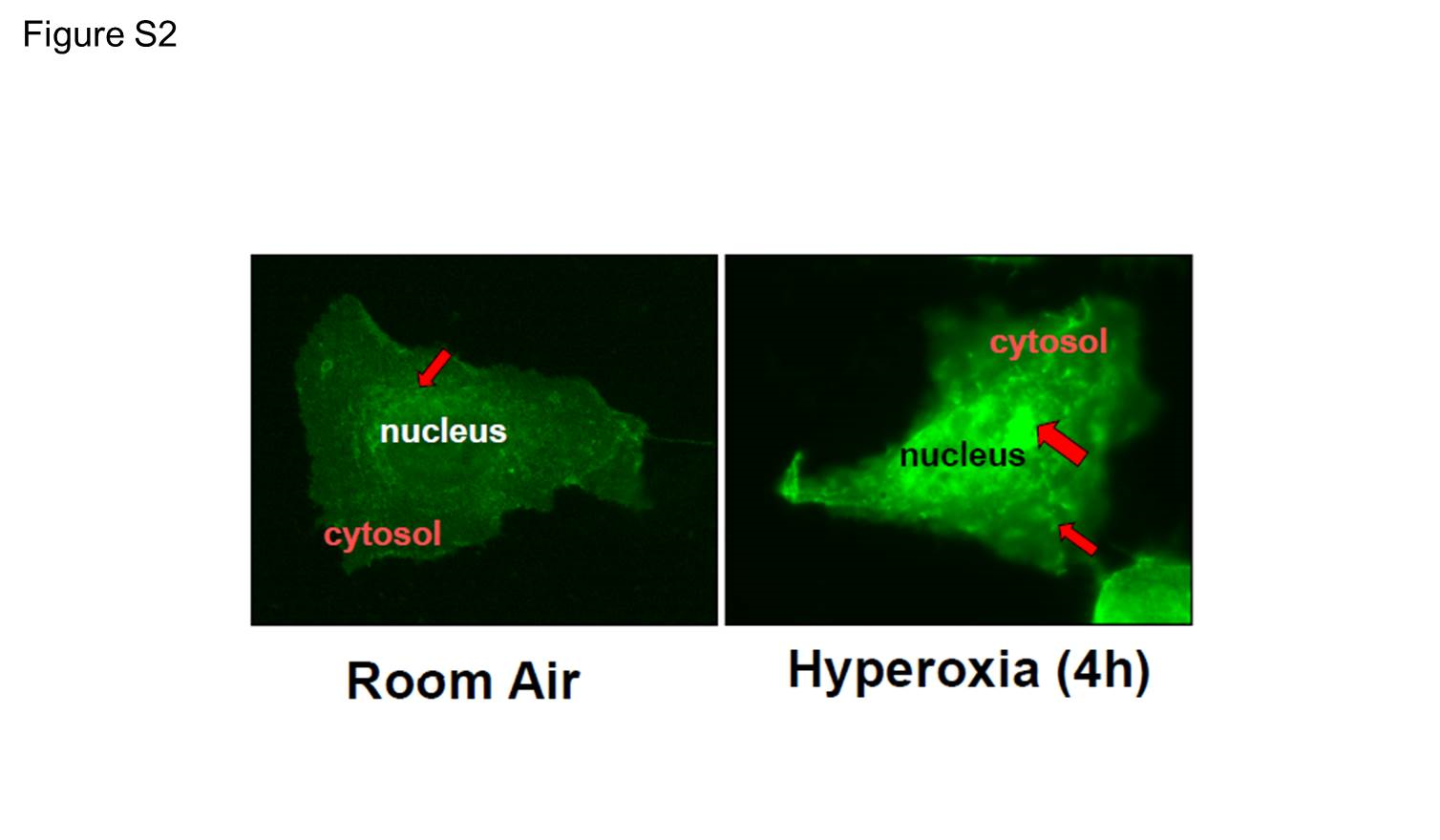

Supplement: Figure S2 — Localization of Flot-2-GFP in Beas2B cells. Cells were exposed to room air and hyperoxia (4h) and the Flot-2 was observed under confocal microscopy. Red arrow: Flot-2. (TIF) [file pone.0077519.s002.tif]

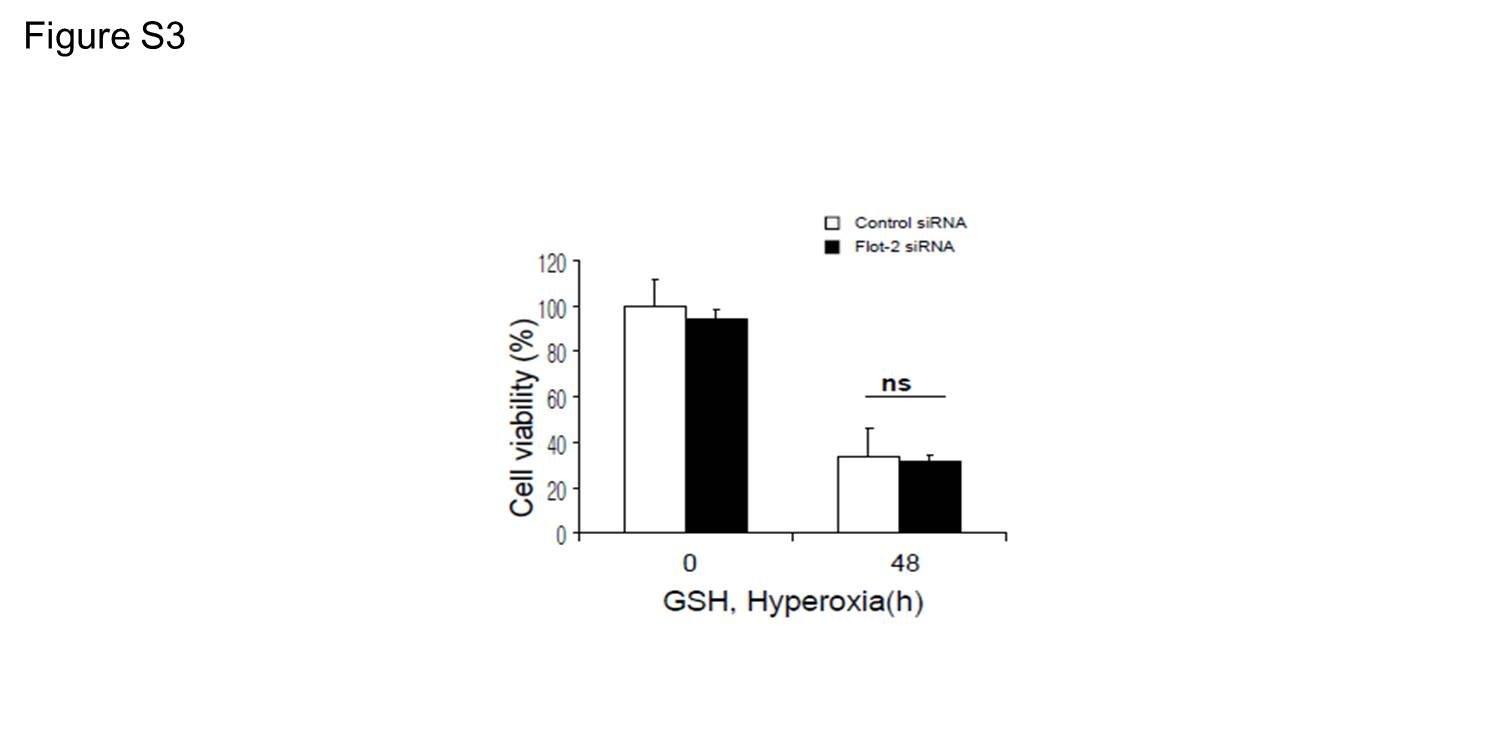

Supplement: Figure S3 — Antioxidant diminished the effects of Flot-2-silencing. Beas 2B cells were transfected with Flot-2 siRNA and control siRNA. Next, cells were treated with antioxidant glutathione (GSH, 80 µM) and exposed to hyperoxia. After 48h, cell viability was performed using CellTiter-Glo Luminescent Cell Viability Assay as described in material and methods. All figures above represented three independent experiments with similar results. ns: non-significant. (TIF) [file pone.0077519.s003.tif]

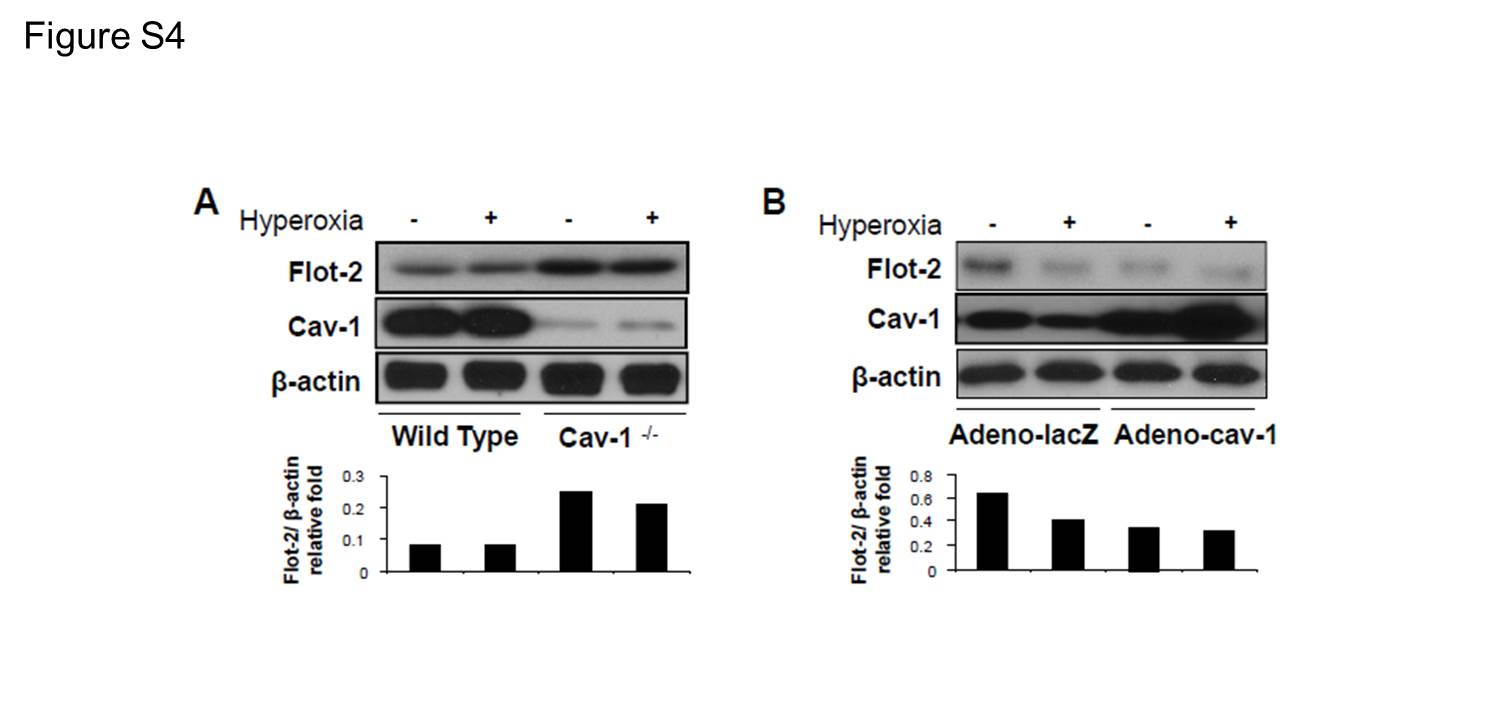

Supplement: Figure S4 — Deletion or enhancement of cav-1 regulates Flot-2 expression. (A) Primary lung epithelial cells were isolated from wild type C57BL/6J mice or cav-1−/− mice. Cells were then exposed to room air or hyperoxia. After 24h, cell lysate was collected and subjected to Western blot analysis. (B) LacZ and adeno-cav-1 transfected to Beas2B cells for 48h. And then Cells were then exposed to room air or hyperoxia for 24h. The figure represented three independent experiments with similar results. (TIF) [file pone.0077519.s004.tif]
